# Efficacy of Moxibustion for Primary Osteoporosis: A Trial Sequential Meta-Analysis of Randomized Controlled Trials

**DOI:** 10.1155/2022/1268876

**Published:** 2022-09-27

**Authors:** Xiang Li, Zihan Yin, Xiao Li, Bingzun Yin, Yixiang Liu, Wenchuan Qi, Fanrong Liang

**Affiliations:** ^1^School of Acu-Mox and Tuina, Chengdu University of Traditional Chinese Medicine, Chengdu, China; ^2^Luohu Hospital of Traditional Chinese Medicine, Shenzhen, China

## Abstract

**Background:**

Primary osteoporosis (PO) is a systemic metabolic skeletal disease. Previous studies have shown that moxibustion can reduce pain intensity and enhance response rate, bone mineral density (BMD), and living function of the patients with PO. However, consensus on its efficacy does not exist, and evidence of moxibustion for PO is also insufficient.

**Methods:**

We searched five English and four Chinese databases with various additional sources and published reviews through December 1, 2021, to evaluate potentially concerned randomized controlled trials (RCTs). Two independent researchers addressed selection screening, data extraction, and risk of bias assessment. The data of this meta-analysis were analyzed using the RevMan v.5.4 software. Additionally, the trial sequential analysis v.0.9.5.10 *β* was used to estimate the sample size. In contrast, the quality of evidence from the RCTs was assessed using the Grading of Recommendations, Assessment, Development, and Evaluation tool.

**Results:**

The current meta-analysis included 14 RCTs containing 898 participants. The methodological quality of the RCTs was moderate. The review demonstrated that a combination of moxibustion and conventional medicine (CM) significantly reduced pain intensity and improved the BMD compared with CM. Furthermore, it was found that moxibustion plus CM/moxibustion could improve response rates compared with CM. However, it was found that the reduction of pain intensity and improvement of BMD by moxibustion showed no significant difference compared with CM. It was also evident that the sample size of most outcomes was inadequate. Moreover, all evidence obtained in this study was ranked as low to critically low.

**Conclusions:**

In conclusion, it was demonstrated that moxibustion is a potentially effective agent for treating PO. However, high-quality studies should be implemented in the future because this study only obtained low-quality evidence. This study was registered in the PROSPERO platform (CRD42021291310).

## 1. Introduction

Primary osteoporosis (PO) is a systemic metabolic skeletal disease that is characterized by reduced bone mineral density (BMD) and microarchitectural [1, 2]. Additionally, PO may cause significant harm, including chronic pain and fragility fractures, which lead to decreased quality of life [1, 2]. PO has become one of the most frequent human diseases and a primary global public health issue with the progressive aging of the population [3]. In 2010, it was reported that the number of adults with osteoporosis was 10.2 million, affecting between 6 and 11% of adults aged 50 years and above, translating to approximately one in every 9–17 adults [4, 5]. Furthermore, the annual total population of facility-related hospitals in the United States costs $5.1 billion [6, 7]. Conversely, the Seventh National Census statistics indicated that the prevalence of PO among Chinese senior citizens over 60 years was approximately 36% [8], with nearly 95 million cases of PO in China. Therefore, because of the growing number of senior citizens worldwide, the management of PO has gained increased attention in many nations [9, 10].

The United States Food and Drug Administration (FDA) has approved multiple pharmacotherapy therapies to treat PO [11]. However, pharmacotherapy has definite limitations in numerous clinical practices [12, 13]. Therefore, there is an urgent need to investigate a new effective nonpharmacological therapy for PO. Moxibustion is a nonpharmacological therapy widely used to manage osteoporosis in China [14–16]. Some previous studies have revealed that moxibustion may alleviate osteoporosis pain, improve BMD, and reduce the response rate, among others. Moreover, some previous studies [17–22] have shown that moxibustion can improve BMD, bone strength, and hormone levels, effectively increasing the vitamin D level in serum. Moxibustion also improves bone calcium content and BMD by regulating calcium and phosphorus metabolism. Thus, the experiments' findings are moderately convincing that moxibustion can benefit osteoporosis.

Based on the available studies, it is evident that some previous systematic reviews (SR) still exist [23]. However, no definite conclusion has been confirmed regarding the efficacy of moxibustion in treating PO [24]. Simultaneously, numerous randomized controlled trials (RCTs) have revealed that moxibustion may be applied for treating PO compared with first-line treatment, including calcium supplementation. Furthermore, the sample size of these RCTs was found to be commonly small, which none of the studies conducting sample size estimation and sequential analysis of the included studies, which could lead to bias and false-positive results. Therefore, this meta-analysis was designed to resolve the described issues and provide evidence regarding moxibustion therapy's efficacy in managing PO.

## 2. Methods

This present review was registered at PROSPERO (CRD42021291310). In addition, the study was conducted according to the guidelines of the Preferred Reporting Items for Systematic Reviews and Meta-Analyses (PRISMA) [25], and a measurement tool to assess systematic Reviews-2 [26].

### 2.1. Eligibility and Exclusion Criteria

#### 2.1.1. Eligibility Criteria

- Types of studies: the study included all randomized controlled parallel trials of moxibustion for PO published, regardless of the language or publication type.
- Types of participants: participants with all types of PO, regardless of type, gender, age, etiology, ethnic groups, severity, and diagnosis with specific criteria, were eligible for inclusion in this review.
- Types of interventions: the moxibustion approach was included in this present review as a monotherapy or complementary therapy.
- Types of the control group: the control group included the conventional-based medicine group (calcium supplementation).
- Types of outcome measures: our primary outcome was a reduction in pain intensity (as determined by the Visual Analogue Scale, VAS). The secondary outcomes included response rate, BMD improvement of the lumbar spine, and improvement in limited mobility (as determined by Oswestry Disability Index, ODI).

### 2.2. Exclusion Criteria

Studies that met any of the following exclusion criteria were excluded in this present review: (1) Non-RCTs, qualitative studies, case reports, conference abstracts, expert experience, letters, comments, animal studies, and duplicated articles; (2) Incomplete research information.

### 2.3. Search Strategy

This review retrieved studies published from inception to December 1, 2021, from various databases, including the Chinese Biomedical Literature Database, China National Knowledge Infrastructure, Wanfang Database, VIP Database, Web of Science (1965–2021) through the Web of Knowledge, Embase (1974–2021) through Ovid, Medical Literature Analysis and Retrieval System Online (1966–2021) through PubMed, the Cochrane Central Register of Controlled Trials (The Cochrane Library, 2021, Issue 8), and Allied and Complementary Medicine Database (1985–2021) through EBSCO.

This study searched for the ongoing trials with unpublished data in the clinical trial registries, including the World Health Organization International Clinical Trials Registry Platform, National Institutes of Health clinical registry (Clinical trials.gov), and the Chinese Clinical Trial Register (ChiCTR), to minimize the risk of publication bias. Moreover, reference lists from similar published SRs/MAs were manually reviewed. The search terms used were osteoporosis, bone loss, brittle-bone disease, moxibustion, acupoint, and random trial, among others. Furthermore, the terms were connected using “and,” “or,” whereas Chinese retrieval modes were similar to English retrieval and the searching strategies of databases, as shown in Appendix 1.

### 2.4. Study Selection and Data Extraction

Two researchers (ZY and XL) were trained to independently extract data for this study. During the study selection, the researchers excluded duplicate studies using NoteExpress V.3.0. Subsequently, they assessed the titles/abstracts of identified studies to exclude unmatched studies according to exclusion criteria. Finally, the researchers read the full text of the studies to select those that met the inclusion criteria for this study. Accordingly, any divergences in the data obtained were solved through discussion between them.

Data extraction was accomplished by the reviewers (ZY and XL) using a standardized data extraction form that included the following information: the first author, publication year, country, sample size, allocation ratio, type of PO, age, gender, course of the disease, moxibustion group detail, control group detail, acupoint, duration of treatment, follow-up period, and outcome. Furthermore, any disagreements were resolved by a third party (XL or LZ) and the original corresponding/first author of the study was contacted in case of missing or incomplete information in any RCTs.

### 2.5. Quality Assessment

The Cochrane risk of bias tool 2.0 (ROB 2.0) [27] was used for the risk of bias. The ROB 2.0 contains five domains (the randomization process, deviation from intended interventions, missing outcomes data, outcome measurement, and selection of the reported results) of low, high, or unclear risk bias. Two researchers (ZY and XL) independently assessed the quality, whereas any disagreement was resolved by an intercessor (LZ).

### 2.6. Statistical Analysis

Statistical analysis of the data obtained in this study was performed using Review Manager (RevMan) Version 5.4 software. Risk ratios (RRs) with a 95% confidence interval (CI) were also calculated for the dichotomous data. In addition, the mean differences with a 95% CI were calculated for continuous data. The fixed-effects model was used for data analysis when the *p* ≥ 0.1 and *I*^2^ ≤ 50%; otherwise, the random-effects model was applied. Moreover, the fixed-effects model was conducted based on the Mantel–Haenszel method. Otherwise, the random-effects model was performed using the Der Simonian-Laired method. In this study, sensitivity analyses were also performed on the data obtained to determine the robustness of the results. Furthermore, when the included RCTs > 2, the potential publication bias of the studies was investigated using Egger's test, and no publication bias was reported if the *p* < 0.05.

### 2.7. Trial Sequential Analysis

TSA [28, 29] was used to reduce the risk of false-positive results by repeating statistical tests and detecting the required information size (RIS) using TSA 0.9.5.10 *β* software (Copenhagen Trial Unit, Centre for Clinical Intervention Research, Copenhagen, Denmark, 2016) for each outcome. This review calculated the RIS for each variable using a value of 5% for type I error and 20% for type II error (equal to 80% power). Therefore, this study displayed futility boundaries according to O'Brien-Fleming's alpha-spending function; the difference between the two therapies demonstrated a sufficient sample size if the cumulative Z-curve exceeded the futility boundaries.

### 2.8. Quality of Evidence

The Grading of Recommendations, Assessment, Development, and Evaluation (GRADE) tool [30] was used to assess the quality of evidence and categorize them as high, moderate, low, or critically low.

### 2.9. Assessing Reviewer Agreements

The intraclass correlation coefficient score [31] of the two researchers in this review study was 0.86.

## 3. Results

### 3.1. Study Description

#### 3.1.1. Literature Search

**Figure 1:**
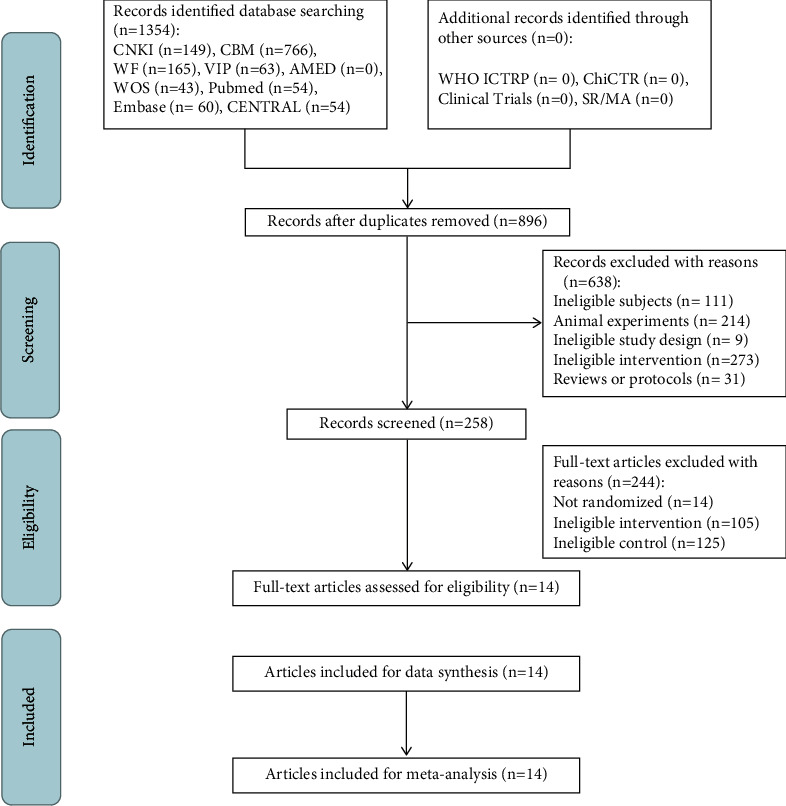
The PRISMA flow chart of selection process.

This study's searching and selecting process is shown in the PRISMA flowchart in Figure 1. Overall, 1354 potentially eligible studies were found after a comprehensive search during this review. Furthermore, 896 articles were identified after removing duplicate records for initial screening. After initial screening, 258 articles were subsequently left for review of their full text. Finally, 244 articles were excluded (14 non-RCT, 105 nonmoxibustion, and 125 noncalcium supplementations) from this review following the full text articles screening, and 14 studies [32–45] with 898 patients remained for the systematic review.

#### 3.1.2. Study Characteristics

The primary characteristics of the 14 articles included in this SR are summarized in Table 1. All included studies were conducted in China, with sample sizes of less than 90. In addition, the allocation ratios of 12 included trials [32–45] were 1 : 1. Postmenopausal osteoporosis is generally considered the most common type of PO. Furthermore, the mean age of the patients was 40 to 80 years, and the proportion of females was significantly higher than that of males. Simultaneously, the mean course of the disease was 20 to 60 months.

In the intervention group, it was found that moxibustion plus calcium supplementation was the frequently used therapy, and the control groups only used calcium supplementation. The intervention duration of 8 trials (57.14%) was 90 days (3 months). However, it was evident that only two studies included follow-up (30 days). In addition, it was found that the most reported outcome was pain intensity improvement. Specific acupoints of the moxibustion therapy are presented in Table 1; Shenshu (BL23) and Mingmen (GV4) were the most frequently used acupoints, whereas the most commonly used meridian was Du Meridian.

### 3.2. Quality Assessment

**Figure 2:**
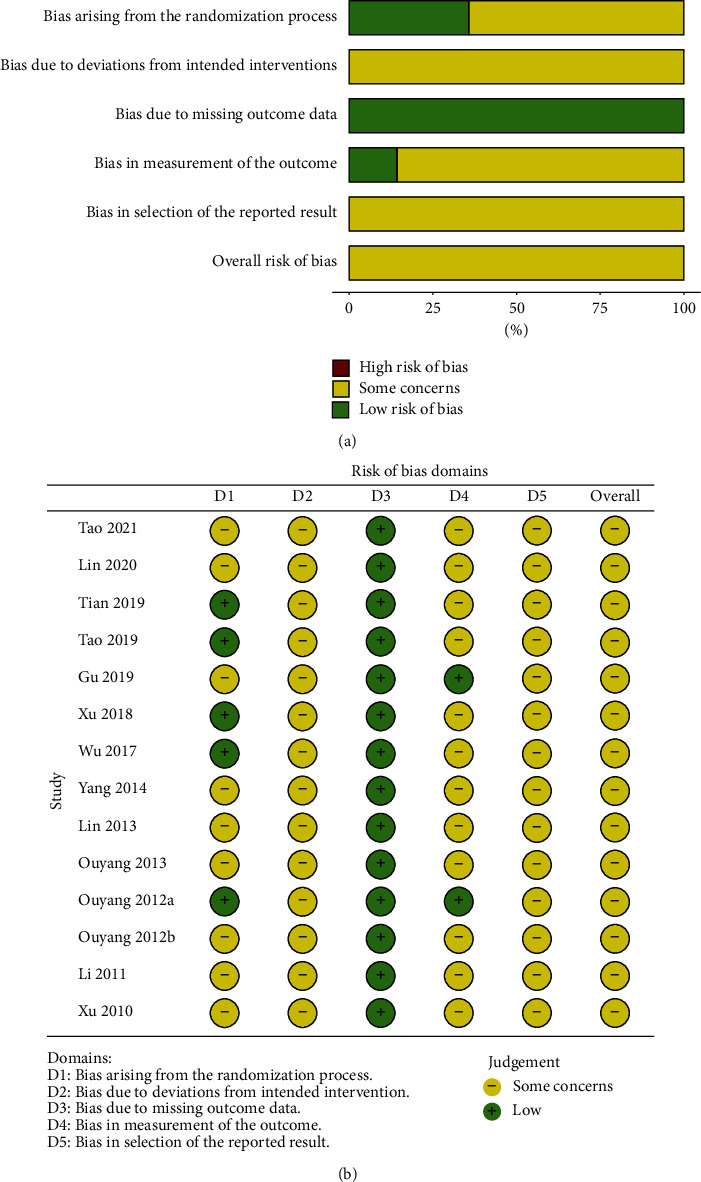
(a) Risk of bias graph. (b) Risk of bias summary.

The ROB of RCTs included in this SR was assessed according to the Cochrane ROB 2.0 tool. In addition, the ROB graphs (Figure 2) were generated using the Shiny app (https://mcguinlu.shinyapps.io/robvis/). Although all included articles were reported being randomized in the randomization process, nine RCTs were uncertain because of unclear random sequence generation. In deviation from the intended interventions, it was found that all studies had the same concerns because of the lack of explanation. Only two studies [35, 43] in this SR demonstrated reliable outcome measurement. Notably, all studies were at a low risk of missing outcome data. Fourteen RCTs were evaluated for some concerns because of the lack of protocol/registration when selecting the reported results.

## 4. Results of Meta-Analysis and TSA

### 4.1. Reduction in Pain Intensity

#### 4.1.1. Moxibustion Vs. CM

**Figure 3:**
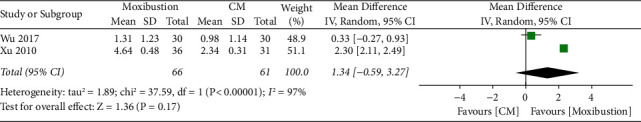
Meta-analysis forest plot of reduction in pain intensity of moxibustion vs. CM.

**Figure 4:**
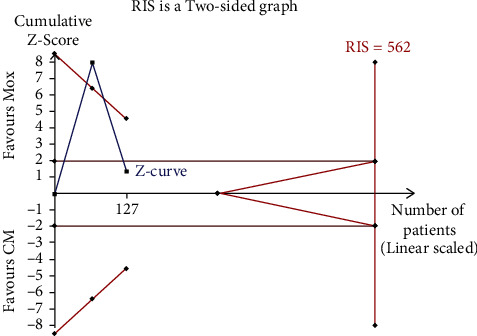
Trial sequential analysis on moxibustion vs. CM of reduction in pain intensity.

Overall, two trials investigated the reduction in pain intensity of moxibustion on PO compared with CM, according to the findings of this study. A random-effects model revealed no significant difference (*n* = 127; MD, 1.34, 95% CI (−0.59; 3.27); Figure 3) in the reduction of pain intensity with marked heterogeneity (*I*^2^ = 97%, *p* < 0.00001). Therefore, various acupoints may result in heterogeneity. In a comparative analysis between moxibustion and CM, TSA revealed that the cumulative Z-curve uncrossed the RIS boundary (RIS = 562), indicating that the sample size was insufficient to confirm the findings of the study (Figure 4).

#### 4.1.2. Moxibustion plus CM vs. CM

**Figure 5:**
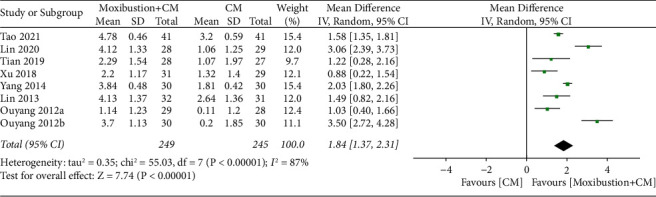
Meta-analysis forest plot of reduction in pain intensity of moxibustion plus CM vs. CM.

**Figure 6:**
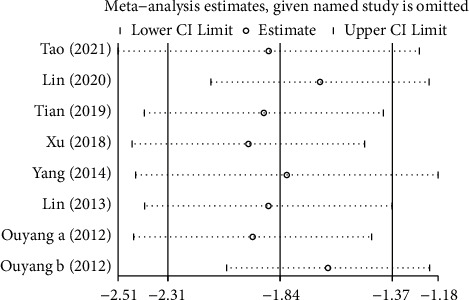
Sensitivity analysis of reduction in pain intensity of moxibustion plus CM vs. CM.

Overall, eight trials investigated the reduction in pain intensity of moxibustion plus CM on PO compared to CM. In addition, the random-effects model revealed a significant difference (*n* = 494; mineral density [MD], 1.84, 95% CI (1.37; 2.31); Figure 5) in the reduction of pain intensity with marked heterogeneity (*I*^2^ = 87%, *p* < 0.00001). A sensitivity analysis was conducted, which clearly demonstrated that the inclusion or exclusion of any article had no substantial impact on the MD estimate (Figure 6).

**Figure 7:**
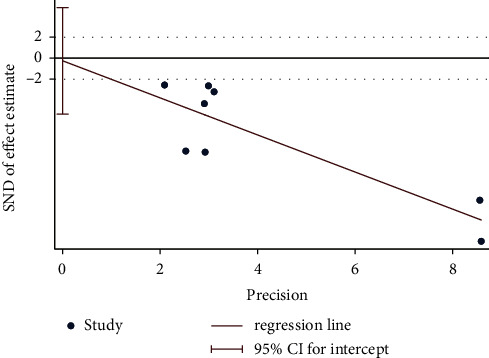
Egger's test plot on moxibustion plus CM vs. CM of reduction in pain intensity.

**Figure 8:**
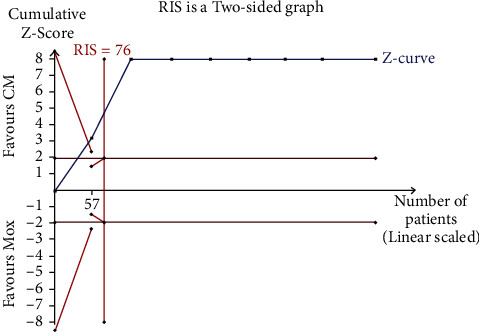
Trial sequential analysis on moxibustion plus CM vs. CM of reduction in pain intensity.

Results of the Egger's test conducted in this study revealed no significant reporting bias among RCTs for improvement in BMD (*p*=0.909 > 0.05; Figure 7). Notably, TSA revealed that the cumulative Z-curve had crossed the RIS boundary (RIS = 76) in a comparative study between moxibustion plus CM and CM, and this indicated that the sample size was sufficient to determine whether moxibustion plus CM was superior to CM in reducing pain intensity (Figure 8).

### 4.2. Response Rate

#### 4.2.1. Moxibustion Vs. CM

**Figure 9:**
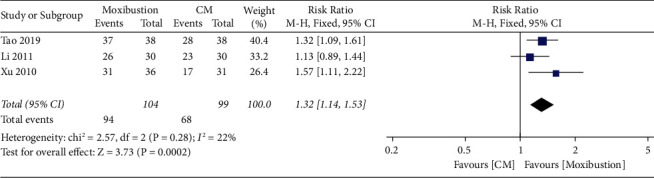
Meta-analysis forest plot of response rate of moxibustion vs. CM.

**Figure 10:**
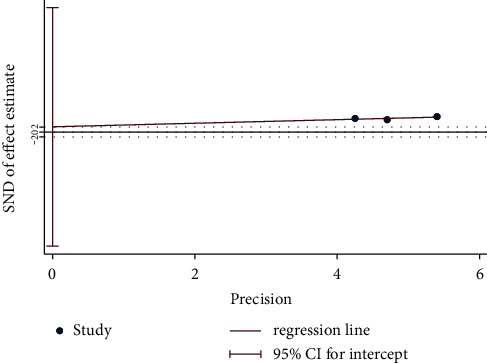
Egger's test plot on moxibustion vs. CM of response rate.

Overall, three trials investigated the response rate of moxibustion on PO compared with CM. A fixed-effect model in this study revealed a significant difference (*n* = 203; RR, 1.32, 95% CI (1.14; 1.53; Figure 9) in the response rate with low heterogeneity (*I*^2^ = 22%, *p*=0.28). In addition, Egger's test results in this review showed no significant reporting bias among RCTs for response rate (*p*=0.679 > 0.05; Figure 10). A comparative analysis between moxibustion and CM noted that TSA revealed an ignored boundary RIS because of insufficient information used.

#### 4.2.2. Moxibustion plus CM vs. CM

**Figure 11:**
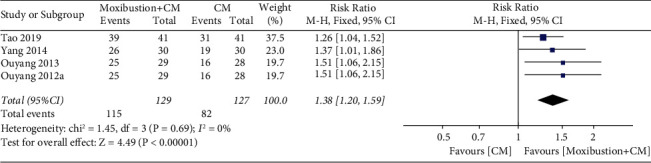
Meta-analysis forest plot of response rate of moxibustion plus CM vs. CM.

**Figure 12:**
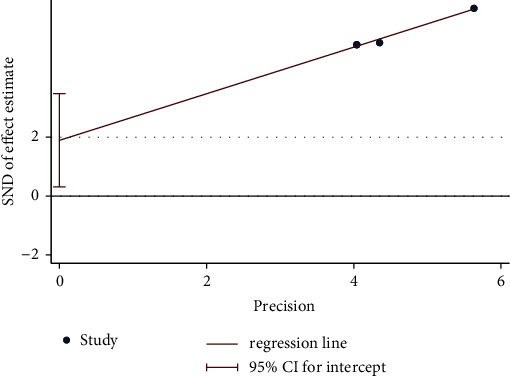
Egger's test plot on moxibustion plus CM vs. CM of response rate.

Overall, four trials investigated the response rate of moxibustion plus CM on PO compared with CM. A fixed-effect model revealed significant difference (*n* = 256; RR, 1.38, 95% CI (1.20; 1.59); Figure 11) in the response rate with low heterogeneity (*I*^2^ = 0%; *p*=0.69). In addition, Egger's test results in this review showed significant reporting bias among RCTs for response rate (*p*=0.036 < 0.05; Figure 12). A comparative analysis between moxibustion plus CM and CM noted that TSA revealed an ignored boundary RIS because of insufficient information used.

### 4.3. Improvement in BMD

#### 4.3.1. Moxibustion vs. CM

**Figure 13:**
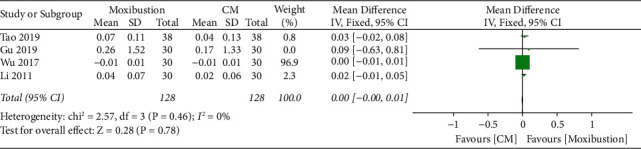
Meta-analysis forest plot of improvement in BMD of moxibustion vs. CM.

**Figure 14:**
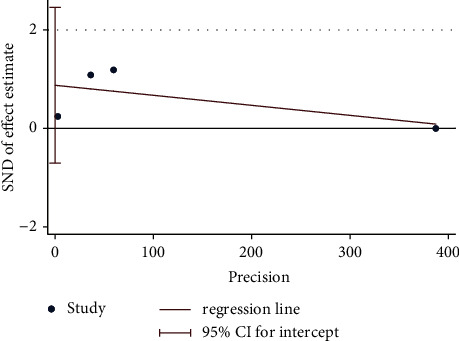
Egger's test plot on moxibustion vs. CM of improvement in BMD.

Overall, four trials investigated the improvement in BMD of moxibustion on PO compared with CM. A fixed-effect model indicated no significant difference (*n* = 256; MD, 0.00, 95% CI (0.00; 0.01; Figure 13) in BMD improvement with low heterogeneity (*I*^2^ = 0%, *p*=0.46). Egger's test showed no significant reporting bias among RCTs for improvement in BMD (*p*=0.140 > 0.05; Figure 14). In a comparative analysis between moxibustion and CM, TSA revealed that boundary RIS was ignored because of insufficient information used.

#### 4.3.2. Moxibustion plus CM vs. CM

**Figure 15:**
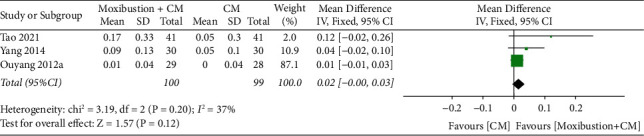
Meta-analysis forest plot of improvement in BMD of moxibustion plus CM vs. CM.

**Figure 16:**
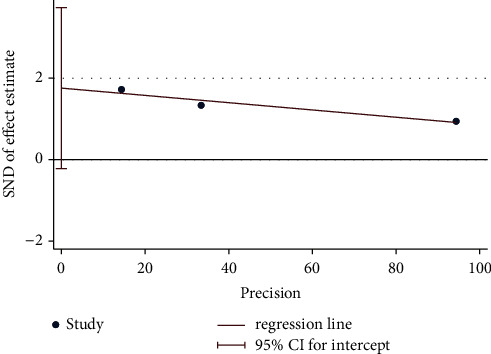
Egger's test plot on moxibustion plus CM vs. CM of improvement in BMD.

Overall, three trials were conducted to compare the improvement in BMD of moxibustion plus CM on PO to CM. A fixed-effect model revealed no significant difference (*n* = 199; MD, 0.02, 95% CI (−0.00; 0.03); Figure 15) in BMD improvement with low heterogeneity (*I*^2^ = 37%, *p*=0.20). Furthermore, the results of Egger's test showed no significant reporting bias among RCTs for improvement in BMD (*p*=0.056 > 0.05) (Figure 16). In a comparative analysis between moxibustion plus CM and CM, TSA revealed that boundary RIS was ignored because of insufficient information used.

### 4.4. Improvement in ODI

**Figure 17:**
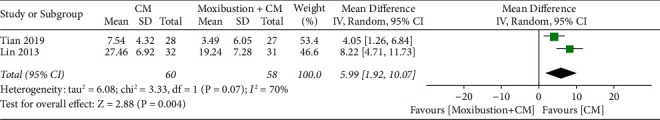
Meta-analysis forest plot of improvement in ODI of moxibustion plus CM vs. CM.

**Figure 18:**
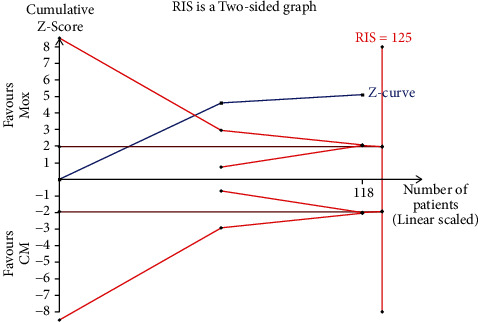
Trial sequential analysis on moxibustion plus CM vs. CM of improvement in ODI.

Overall, two trials investigated the improvement in ODI of moxibustion plus CM on PO compared with CM. The results of a random-effects model showed a significant difference (*n* = 118; MD, 5.99, 95% CI (1.92; 10.07); Figure 17) in ODI improvement with marked heterogeneity (*I*^2^ = 70%, *p*=0.07). Therefore, it was evident that various acupoints may result in heterogeneity. In a comparative analysis between moxibustion plus CM and CM, TSA revealed that the cumulative Z-curve uncrossed the RIS boundary (RIS = 125). Therefore, this indicated that the sample size was insufficient to determine whether moxibustion plus CM was superior to CM regarding ODI improvement (Figure 18).

### 4.5. Safety

Four RCTs reported the safety of the intervention. One study [33] mentioned that moxibustion caused blisters, which did not affect the overall process after proper treatment; another study [44] stated that calcium supplementation caused mild nausea and vomiting; two other studies [34, 45] clearly demonstrated that no adverse effects occurred during the treatment process. The other 10 RCTs did not clearly indicate moxibustion's side effects or safety.

### 4.6. Quality of Evidence

The GRADE approach was used to evaluate the quality of included evidences of the RCTs (Table 2). Four outcomes were included: reduction in pain intensity, response rate, improvement in BMD, and improvement in ODI. Overall, the evidence was of low or critically low quality. However, the poor methodological quality and insufficient sample size were the primary reasons for its degradation.

## 5. Discussion

PO is one of the defining health issues for patients, families, and society of an aging population [1]. Currently, calcium supplementation is the most frequent and recognized antiosteoporosis medicine for treating PO because it improves bone metabolism and BMD [37]. In China, moxibustion has been widely used for several chronic musculoskeletal disorders, including PO and knee osteoarthritis, among others. However, previous studies have generally demonstrated no definite conclusion on the efficacy of moxibustion for PO.

Recently, several clinical RCTs have evaluated the effects of moxibustion on PO, and it has been demonstrated that moxibustion may increase patient benefit against PO. In addition, numerous experiments have shown that moxibustion treatment is associated with increased bone formation markers [41], decreased bone resorption markers c, and increased hormone levels [32] in patients with. Qian and Fan [46] demonstrated that moxibustion treatment could improve the messenger ribonucleic acid (mRNA) level of osteoprogenitor (OPG), reduce the mRNA level of the receptor activator for nuclear factor-*κ*B ligand, promote the combination of OPG and receptor activator for nuclear factor-*κ*B, block signal transmission of osteoclast chain reactions, and inhibit bone resorption. Yao et al. [47] found that moxibustion in specific acupoints in osteoporosis model rats can improve the activity of bone marrow mesenchymal stem cells (BMMSCS), activate the Wnt/*β*-catenin signaling pathway, and promote BMMSCS differentiation into osteoblasts. Therefore, based on the above research evidences, this study was conducted and aimed to provide evidence for the efficacy of moxibustion on PO.

In the 14 RCTs included in this study, moxibustion or moxibustion plus CM (calcium supplementation) was found to be superior to CM. This current review involved four outcomes, including a reduction in pain intensity, response rate, improvement in BMD, and improvement in ODI, and the following findings were obtained: first, it was evident that moxibustion showed no statistically significant difference in the reduction of pain intensity in patients with PO as compared with that of CM. The quality of evidence was classified as critically low. Furthermore, the reduction of pain intensity by moxibustion plus CM showed statistically significant differences as compared with that of CM, and the quality of evidence was classified as low. Second, moxibustion/moxibustion plus CM demonstrated significant differences in response rate as compared with the rate shown by CM, and the evidence quality was ranked as low to critically low. Third, when moxibustion plus CM was compared with CM alone, the results showed a significant improvement in ODI, and the evidence level was classified as critically low. Fourth, it was evident that no statistical differences were reported in increasing BMD between treatments with moxibustion/moxibustion plus CM, as compared with CM only, and the evidence obtained was ranked as low quality. Simultaneously, only one study reported the adverse events caused by moxibustion. To our knowledge, the burning of moxa does not directly contact with the skin of patients, and the treatment process is safe under the close attention of doctors.

To the best of our best knowledge, this study is the first trial sequential meta-analysis to certify moxibustion therapy for reduction in pain intensity, response rate, improvement in BMD, and improvement in ODI in patients with PO. Therefore, this review's findings provide credible evidence to assist in making clinical decisions on the management of PO. Furthermore, a sequential trial analysis was conducted to measure statistical reliability and estimate the optimum sample size of included RCTs. In addition, the GRADE approach was used to evaluate the quality of evidence derived from the included RCTs.

However, this review had some limitations. First, the primary limitation was the low quality of the evidence. Second, the methodological quality of the included studies was poor, which could impact the findings' reliability and reduce efficiency. Third, variability in study design, bias risk, and other factors may cause methodological heterogeneity. Fourth, moxibustion differs in terms of acupoints, method, and frequency, leading to heterogeneity. Fifth, all RCTs published in English/Chinese were implemented in China, which may have resulted in region bias. Sixth, the incidence of fracture, and serum factors (inflammatory indicators, bone metabolism parameters, and other factors) as endpoint outcomes of PO, were ignored in the included studies. Finally, the long-term follow-up evaluation is lacking in this review, yet PO is a chronic disease.

This review identifies some suggestions for further research. First, the overall reliability of the research conclusion is not high because of the poor quality of evidence. Therefore, it is suggested that future research should discreetly and strictly strive to perfect its designs according to the latest edition of the Cochrane Handbook for Systematic Reviews, Consolidated Standards of Reporting Trials [48], and Standards for Reporting Interventions in Clinical Trials of moxibustion [49].

Simultaneously, clinical heterogeneity existed in this review due to factors, including acupoints, and treatment duration, among others. However, this study also clearly had no certain relationships; thus, the relationship between moxibustion factors and efficacy is an important point for future research. In addition, this study aimed to investigate the short-term efficacy of moxibustion; however, the long-term efficacy of moxibustion for PO is yet to be investigated. Therefore, future research should focus on a long-term follow-up period to evaluate the long-term effects of the treatment. Moreover, underlying mechanisms of moxibustion against PO should further be investigated. Finally, fracture incidence is obviously a vital endpoint outcome of PO, and future trials should pay more attention to fracture incidence.

## 6. Conclusion

In conclusion, this study demonstrated that moxibustion can provide clinical benefits for PO conditions and had a significant response rate in PO management. However, due to evidence of poor quality, further research is warranted with additional well-designed and high-quality large-scale RCTs to confirm the findings of this study.

## Figures and Tables

**Table 1:** Main characteristics of included RCTs.

Study	Country	Sample size	Allocation ratio	Type of PO	Age (Year)	Gender (M: F)	Course of the disease (Month)	(A)	(B)	Acupoints selection	Duration of treatment	Follow-up	Efficacy criteria
								Treatment Group	Control Group I				
Tao et al. [32]	China	82	1 : 1	SOP	A: 65.3 ± 1.5 B: 64.5 ± 1.3	A: 19: 22 B: 21: 20	A: 24–48 B: 24–60	Mox + CM	CM (calcium supplementation)	Dazhu (BL11), Shenshu (BL23), Pishu (BL20), Zusanli (ST36), Zhongwan (CV12), Guanyuan (CV4)	90 d	—	1. Response rate 2. BMD 3. VAS

Lin et al. [33]	China	60	1 : 1	PMOP	A: 59.50 ± 5.92 B: 60.75 ± 5.09	All female	—	Mox + CM	CM (calcium supplementation)	Du Meridian, from Dazhui (GV14) to Yaoshu (GV2)	90 d	—	1. VAS

Tian [34]	China	60	1 : 1	PO	A: 64.21 ± 6.50 B: 63.59 ± 6.95	A: 28: 0 B: 26: 1	A: 22.81 ± 12.30 B: 21.89 ± 11.89	Mox + CM	CM (calcium supplementation)	Lumbosacral portion from L2 to S1, Pishu (BL20), Shenshu (BL23), Yaoyangguan (GV3), Mingmen (GV4)	28 d	30 d	1. VAS 2. ODI

Tao [35]	China	76	1 : 1	PO	A: 69.42 ± 6.27 B: 70.38 ± 6.46	A: 22: 16 B: 23: 15	—	Mox	CM (calcium supplementation)	Dazhu (BL11), Shenzhu (GV12), Mingmen (GV4), Shenshu (BL23), Pishu (BL20), Zhongwan (CV12), Shenque (CV8), Zusanli (ST36), Guanyuan (CV4), Xuanzhong (GB39), Sanyinjiao (SP6), Yinlingquan (SP9), Taixi (KI3)	84 d	—	1. Response rate 2. BMD

Gu [36]	China	60	1 : 1	PMO	A: 60.12 ± 3.67 B: 59.65 ± 3.84	Allfemale	A: 54.26 ± 3.58 B: 52.78 ± 4.05	Mox	CM (calcium supplementation)	Shenshu (BL23)	140 d	—	1.BMD

Xu et al. [37]	China	63	32 : 31	PMO	A: 65.16 ± 6.82 B: 63.90 ± 7.59	Allfemale	A: 11.31 ± 4.03 B: 11.32 ± 4.06	Mox + CM	CM (calcium supplementation)	Pishu (BL20), Shenshu (BL23), Yaoyangguan (GV3), Mingmen (GV4)	28 d	30 d	1. VAS

Wu [38]	China	60	1 : 1	PMO	45–65	Allfemale	>12	Mox	CM (calcium supplementation)	Pishu (BL20), Shenshu (BL23)	90 d	—	1. BMD 2. VAS

Yang et al. [39]	China	60	1 : 1	PO	A: 45–80 B: 46–78	A: 11: 19 B: 12: 18	A: 24–84 B: 30–78	Mox + CM	CM (calcium supplementation)	Du Meridian, from Dazhui (GV14) to Yaoshu (GV2)	84 d	—	1. Response rate 2. BMD 3. VAS

Lin et al. [40]	China	70	1 : 1	PMO	45–75	Allfemale	—	Mox + CM	CM (calcium supplementation)	Du Meridian, from Dazhui (GV14) to Yaoshu (GV2)	90 d	—	1. VAS 2. ODI

Ouyang and Xu [41]	China	60	1 : 1	PMO	62.45 ± 7.68	Allfemale	—	Mox + CM	CM (calcium supplementation)	Pishu (BL20), Weishu (BL21), Shenshu (BL23), Mingmen (GV4), Yaoyangguan (GV3), Zhiyang (GV9)	90 d	—	1. Response rate

Ouyang [42]	China	60	1 : 1	PMO	A: 62.29 ± 7.18 B: 61.14 ± 7.68	Allfemale	60	Mox + CM	CM (calcium supplementation)	Dazhu (BL11), Geshu (BL17), Ganshu (BL18), Shenshu (BL23), Pishu (BL20), Mingmen (GV4), Zusanli (ST36), Yanglingquan (GB34), Taixi (KI3), Guanyuanshu (BL26)	90 d	—	1. Response rate 2. BMD 3. VAS

Ouyang et al. [43]	China	60	1 : 1	PO	A: 63.25 ± 10.14 B: 60.11 ± 11.35	A: 12: 18 B: 13: 17	—	Mox + CM	CM (calcium supplementation)	Dazhu (BL11), Geshu (BL17), Ganshu (BL18), Shenshu (BL23), Pishu (BL20), Mingmen (GV4), Zusanli (ST36), Yanglingquan (GB34), Taixi (KI3), Guanyuanshu (BL26)	90 d	—	1. VAS

Li et al. [44]	China	60	1 : 1	PO	40–70	A: 12: 18 B: 10: 20	A: 58.92 ± 36.24 B: 60.72 ± 30.12	Mox	CM (calcium supplementation)	Mingmen (GV4), Shenshu (BL23), Zusanli (ST36), Pishu (BL20)	117 d	—	1. Response rate 2. BMD

Xu and Jin [45]	China	67	36 : 31	PO	50–82	A: 8: 23 B: 9: 27	A: 87.96 ± 44.04 B: 83.64 ± 33.48	Mox	CM (calcium supplementation)	Shenque (CV8), Guanyuan (CV4), Qihai (CV6), Mingmen (GV4), Shenshu (BL23), Pishu (BL20)	90 d	—	1. Response rate 2. VAS

Note: SO: senile osteoporosis; PMO: postmenopausal osteoporosis; PO: primary osteoporosis; Mox: moxibustion; CM: conventional medicine; d: day; VAS: visual analogue scale; BMD: bone mineral density; ODI: Oswestry disability index.

**Table 2:** Quality of evidence included RCTs by GRADE.

Outcomes	Included RCTs (patients)	Relative effect (95% CI)	Quality assessment					Quality of evidence
			Risk of bias	Inconsistency	Indirectness	Imprecision	Publication bias	
Reduction in pain intensity								

Mox vs. CM	2 (127)	MD -1.34 (−3.27 to 0.59)	−1①	−1②	0	−1③	0	Critically low
Mox plus CM vs. CM	8 (494)	MD -1.84 (−2.31 to -1.37)	−1①	−1②	0	0	0	Low

Response rate								

Mox vs. CM	3 (203)	RR 1.32 (1.14 to 1.53)	−1①	0	0	−1③	0	Low
Mox plus CM vs. CM	4 (256)	RR 1.38 (1.20 to 1.59)	−1①	0	0	−1③	−1④	Critically low

Improvement in BMD								

Mox vs. CM	4 (256)	MD 0.00 (0.00 to 0.01)	−1①	0	0	−1③	0	Low
Mox plus CM vs. CM	3 (199)	MD 0.02 (0.00 to 0.03)	−1①	0	0	−1③	0	Low

Improvement in ODI								

Mox plus CM vs. CM	2 (118)	MD -5.99 (-10.07 to -1.92)	−1①	−1②	0	−1③	0	Critically low

Notes: Mox: moxibustion; CM: conventional medicine; BMD: bone mineral density; ODI: Oswestry disability index; MD: mean difference; RR: relative risk; ①Poor methodological quality. ②The size and direction of the effect size, the overlap of the confidence interval is small, the p value of the heterogeneity test is small, and the combined results of I2 value are large. ③Insufficient sample size. ④Significant reporting bias.

## Data Availability

The data would be made available upon request.

## Supplementary Materials

Supplementary Materials Appendix 1. Search strategies of each database.
