# Supplementary material for: Efficacy of Moxibustion for Primary Osteoporosis: A Trial Sequential Meta-Analysis of Randomized Controlled Trials
Source: Evid Based Complement Alternat Med. 2022 Sep 27;2022:1268876. doi: 10.1155/2022/1268876 (PMC9532117; doi:10.1155/2022/1268876)
Supplement: Supplementary Materials — Appendix 1. Search strategies of each database. [file 1268876.f1.docx]

**Appendix 1. Search strategies of each database.**

**PubMed**

#1 "Osteoporosis"[Mesh]

#2 "osteoporosis" [Title/Abstract] OR "bone loss" [Title/Abstract] OR "brittle-bone disease" [Title/Abstract] OR "OP" [Title/Abstract] OR "primary osteoporosis" [Title/Abstract] OR "POP" [Title/Abstract]

#3 #1 OR #2

#4 "Moxibustion"[Mesh]

#5 "acupuncture-moxibustion"[Title/Abstract] OR "meridian*"[Title/Abstract] OR "acupoint*"[Title/Abstract] OR "warm needling"[Title/Abstract] OR "warm acupuncture"[Title/Abstract] OR "acupuncture plus moxibustion"[Title/Abstract] OR "moxibustion"[Title/Abstract]

#6 #4 OR #5

#7 "clinical trials, randomized"[Mesh Terms] OR "controlled clinical trials, randomized"[Mesh Terms] OR "clinical trials as topic"[MeSH Terms] OR "random allocation"[MeSH Terms] OR "therapeutic use"[MeSH Subheading]

#8 ("clinical"[Title/Abstract] AND "trial"[Title/Abstract]) OR "clinical trial"[Publication Type] OR "random*"[Title/Abstract]

#9 #7 OR #8

#10 #3 AND #6 AND #9

**EMBASE**

#1 'Osteoporosis'/exp/mj

#2 'osteoporosis':ti,ab,kw OR 'bone loss':ti,ab,kw OR 'brittle-bone disease':ti,ab,kw OR 'OP':ti,ab,kw OR 'primary osteoporosis':ti,ab,kw OR 'POP':ti,ab,kw

#3 #1 OR #2

#4 'moxibustion'/exp/mj

#5 'acupuncture moxibustion':ti,ab,kw OR meridian*:ti,ab,kw OR acupoint*:ti,ab,kw OR 'warm needling':ti,ab,kw OR 'warm acupuncture':ti,ab,kw OR 'acupuncture plus moxibustion':ti,ab,kw OR moxibustion:ti,ab,kw

#6 #4 OR #5

#7 'clinical trials, randomized'/exp/mj OR 'controlled clinical trials, randomized'/exp/mj OR 'clinical trials as topic'/exp/mj OR 'random allocation'/exp/mj

#8 clinical:ti,ab,kw OR trial:ti,ab,kw OR random*:ti,ab,kw

#9 #7 OR #8

#10 #3 AND #6 AND #9

**Web of science**

TS=(‘osteoporosis’ OR ‘bone loss’ OR ‘brittle-bone disease’ OR ‘OP’ OR ‘primary osteoporosis’ OR ‘POP’) AND TS=(‘acupuncture-moxibustion’ OR ‘meridian*’ OR ‘acupoint*’ OR ‘warm needling’ OR ‘warm acupuncture’ OR ‘acupuncture plus moxibustion’ OR ‘moxibustion’ ) AND TS=(‘random*’ OR ‘clinical’ OR ‘trial’ )

**Cochrane Library**

#1 Mesh descriptor: [Osteoporosis]explode all trees

#2 osteoporosis:ti,ab,kw OR bone loss:ti,ab,kw OR brittle-bone disease:ti,ab,kw OR OP:ti,ab,kw OR primary osteoporosis:ti,ab,kw OR POP:ti,ab,kw

#3 #1 OR #2

#4 Mesh descriptor: [moxibustion] explode all trees;

#5 acupuncture moxibustion:ti,ab,kw OR meridian*:ti,ab,kw OR acupoint*:ti,ab,kw OR warm needling:ti,ab,kw OR warm acupuncture:ti,ab,kw OR acupuncture plus moxibustion:ti,ab,kw OR moxibustion:ti,ab,kw

#6 #4 OR #5

#7 Mesh descriptor: [clinical trials, randomized] or [controlled clinical trials, randomized] or [clinical trials as topic] or [random allocation] explode all trees;

#8 clinical:ti,ab,kw OR trial:ti,ab,kw OR random*:ti,ab,kw

#9 #7 OR #8

#10 #3 AND #6 AND #9

**CNKI**

TKA=(‘灸’) AND TKA=(‘骨质疏松’+‘骨质酥松’+‘骨密度’+‘骨质丢失’) AND TKA=(‘随机’+‘对照’)

**WF**

题名或关键词:(骨质疏松 OR 骨质酥松 OR 骨密度 OR 骨质丢失) and 题名或关键词:(灸) and 题名或关键词:(对照 OR 随机)

**Chongqing VIP**

M=(骨质疏松 OR 骨质酥松 OR 骨密度 OR 骨质丢失) and M=(灸) and R=(随机 OR 对照)

**CBM**

1 "骨质疏松" [加权:扩展]

2 "骨质疏松"[常用字段:智能] OR "骨质酥松"[常用字段:智能] OR "骨密度"[常用字段:智能] OR "骨质丢失"[常用字段:智能]

3 1 OR 2

4 "针灸疗法"[加权:扩展] OR "灸法"[加权:扩展] OR "温针疗法"[加权:扩展]

5 "灸"[常用字段:智能] OR "温针"[常用字段:智能] OR "针灸"[常用字段:智能] OR "穴"[常用字段:智能] OR "经络"[常用字段:智能]

6 4 OR 5

7 "随机对照试验"[不加权:扩展]

8 "随机"[常用字段:智能] OR "对照"[常用字段:智能]

9 7 OR 8

10 3 AND 6 AND 9
